# In-Depth Mapping of the Urinary *N*-Glycoproteome: Distinct Signatures of ccRCC-related Progression

**DOI:** 10.3390/cancers12010239

**Published:** 2020-01-18

**Authors:** Lucia Santorelli, Giulia Capitoli, Clizia Chinello, Isabella Piga, Francesca Clerici, Vanna Denti, Andrew Smith, Angelica Grasso, Francesca Raimondo, Marco Grasso, Fulvio Magni

**Affiliations:** 1 Clinical Proteomics and Metabolomics Unit, School of Medicine and Surgery, University of Milano-Bicocca, 20854 Vedano al Lambro, Italy; clizia.chinello@gmail.com (C.C.); isabella.piga@unimib.it (I.P.); f.clerici10@campus.unimib.it (F.C.); v.denti@campus.unimib.it (V.D.); andrew.smith@unimib.it (A.S.); francesca.raimondo@unimib.it (F.R.); fulvio.magni@unimib.it (F.M.); 2 Centre of Biostatistics for Clinical Epidemiology, School of Medicine and Surgery, University of Milano-Bicocca, 20854 Vedano al Lambro, Italy; g.capitoli@campus.unimib.it; 3 Urology Service, Department of Surgery, EOC Beata Vergine Regional Hospital, 23, 6850 Mendrisio, Switzerland; angelica_grasso@yahoo.it; 4 Urology Unit, S. Gerardo Hospital, 20900 Monza, Italy; m.grasso@hsgerardo.org

**Keywords:** clear cell Renal Cell Carcinoma, urine, glycoproteomics, *N-*glycomapping, label-free, glycomarkers

## Abstract

Protein *N*-glycosylation is one of the most important post-translational modifications and is involved in many biological processes, with aberrant changes in protein *N*-glycosylation patterns being closely associated with several diseases, including the progression and spreading of tumours. In light of this, identifying these aberrant protein glycoforms in tumours could be useful for understanding the molecular mechanism of this multifactorial disease, developing specific biomarkers and finding novel therapeutic targets. We investigated the urinary *N*-glycoproteome of clear cell renal cell carcinoma (ccRCC) patients at different stages (*n* = 15 at pT1 and *n* = 15 at pT3), and of non-ccRCC subjects (*n* = 15), using an *N*-glyco-FASP-based method. Using label-free nLC-ESI MS/MS, we identified and quantified several *N*-glycoproteins with altered expression and abnormal changes affecting the occupancy of the glycosylation site in the urine of RCC patients compared to control. In particular, nine of them had a specific trend that was directly related to the stage progression: CD97, COCH and P3IP1 were up-expressed whilst APOB, FINC, CERU, CFAH, HPT and PLTP were down-expressed in ccRCC patients. Overall, these results expand our knowledge related to the role of this post-translational modification in ccRCC and translation of this information into pre-clinical studies could have a significant impact on the discovery of novel biomarkers and therapeutic target in kidney cancer.

## 1. Introduction

Malignant transformation is a complex of heterogeneous cellular events that regulates the growth and survival cycle of affected cells. Among cancer-associated alterations, changes in protein *N*-glycosylation have recently received attention as one of the key events that are able to influence the onset of neoplasia and its consequent spreading [1].

*N*-glycosylation represents one of the most prominent protein post-translational modification (PTMs) and play a role in determining several protein properties, defining its correct tertiary structure, specific function as well as its cellular localisation [2]. Under physiological conditions, *N*-glycosylated proteins have various biological functions, including folding and quality control, cell adhesion and motility, molecular trafficking, cell signalling, immune recognition and clearance [3].

Moreover, the fundamental role of this PTM in oncogenesis has been widely documented over seven decades [4,5]. In fact, high levels of altered *N*-glycosylated proteins are demonstrated to promote tumour invasiveness as well as being correlated with a high frequency of tumour recurrence and metastasis. Furthermore, aberrant changes to *N*-glycosylated protein patterns frequently accompany the transformation of normal tissue towards neoplasia [6,7] and these abnormal changes essentially affect the occupancy of the glycosylation sites (glycan macro-heterogeneity) and/or the attached *N*-glycan structures (glycan micro-heterogeneity) [8,9].

Given their evident role in cancer development, a consistent group of glycoproteins have recently passed the discovery and validation phases and are regularly used in clinical practice as cancer biomarkers, successfully completing the research bench to the patient bedside program. Most of the approved cancer glycoprotein-based biomarkers (glycomarkers) are proteins derived from diverse bodily fluids. Prostate-specific antigen (PSA), for example, which is largely present in seminal fluids and plasma, has been used to screen and monitor prostate cancer patients for 20 years [10,11]. Cancer antigen 15-3 and cancer antigen 125, both detected in serum [12], are useful biomarkers for breast [12] and ovarian [13,14] tumours, respectively, and are particularly used for monitoring affected patients and evaluating the possibility of disease recurrence. Finally, *N*-glycosylated protein markers also include the carcinoembryonic antigen (CEA), detectable principally in blood [15,16], which has been correlated with colorectal, bladder, breast, pancreatic and lung cancers [17,18].

In this context, an easily accessible biological sample, such as urine, is a valuable source of glycomarkers for kidney cancer-related diseases. Urine can be collected non-invasively and in large quantities, with its molecular composition being less complex than other bodily fluids, and it is for these reasons that the use of the urinary proteomics has expanded exponentially in recent years [19,20]. Owing to the rapid development of MS-based technologies and their application in clinical research, the urinary proteome was extensively explored and numerous urinary glycoproteins were brought to light and characterised in healthy subjects [21]. Concerning, the typical proteome of healthy human urine counts almost 2500 proteins [22] and about 300 of these are reported to be *N*-glycosylated [23].

Whilst the urine proteome was widely investigated in clear cell renal cell carcinoma (ccRCC), the most aggressive RCC morphotype [24], limited information describing the urinary *N*-glycoproteome is available. Furthermore, despite ccRCC being commonly diagnosed at the early stages, its aggressiveness and clinical outcomes remain heterogeneous within each staging group, making the research for novel diagnostic and prognostic predictors an urgent priority.

Since cancer transformation causes alterations in the synthesis and expression of specific glycosylated proteins, evaluating the urinary glycoprotein content of ccRCC-affected patients represents a valid strategy to expand our knowledge regarding the role of this modification in the onset of cancer. In this context, we applied a glycoproteomic approach to study urine of ccRCC patients at early (pT1) and advanced (pT3) stages compared to urine of unaffected ccRCC subjects (controls, CTRL). Our final goal was to identify potential biologically relevant indicators of the development and progression of ccRCC in order to provide support for stage-related classification and defining the course of treatment.

## 2. Results

### 2.1. Clinical Data and Study Design

To identify potential protein *N*-glycoforms of interest, urine of 15 controls and 30 ccRCC patients were collected. Three urine pools were prepared, one representative of the CTRL and other two of ccRCC, one of pT1 and one of pT3 (15 subjects for each pool) (Table 1). Then, each pool was analysed by nUHPLC-MS/MS.

### 2.2. Mapping of the N-Glycosylation Sites

Urinary *N*-glycoproteins were extracted by an *N*-Glyco-FASP method [25]. Tryptic peptides (~100 µg) were incubated in the presence of a triad of lectins that are able to bind to different types of carbohydrates. By using filter units, the *N*-glycosylated peptides were then isolated and underwent enzymatic hydrolysis by PNGase F. This enzymatic reaction caused the release of the deglycosylated peptides from lectins and were then analysed by nanoLC-ESI-MS/MS. Those peptides that were present in the whole proteome and not deamidated as a result of the PNGase F treatment were removed from the list of the *N*-glycopeptides.

First, we investigated the whole *N*-glycopeptidome of the three groups of patients in order to uncover possible differences in terms of *N*-glycan macro-heterogeneity. More than 760 *N*-glycopeptides were comprehensively identified: 479 in CTRL, 484 in pT1 and 513 in pT3, respectively (Table S1). Among these, 35.1% were observed to be present in all the groups, 24% were exclusively present in two of the three groups (CTRL and pT1 3%; CTRL and pT3 10.7%, pT1 and pT3 10.3%), whilst the 14.2%, 15.3% and 11.4% were specifically related to CTRL, pT1 and pT3, respectively.

In order to determine the recurrence frequency of the consensus and non-consensus regions in the identified peptides, we subsequently evaluated the sites distribution of the *N*-glycosylation in the urinary proteome. Considering that the canonical *N*-glycosylation motif of proteins is N-x-S/T (where x are all the amino acids, with the exception of proline), we observed a variation of the glycosylated sites between the CTRL and ccRCC patients (Table 2a).

Initially, we compared the CTRL and the ccRCC subjects, without considering the pT feature as variable. We observed that the ratio between the number of asparagine modified (N) present in the glycopeptides-enriched fraction and the total number of asparagine, modified and unmodified (N) in the corresponding proteome was constant in the two groups. For both samples, threonine (T) and serine (S) were significantly overrepresented, meaning that most of the identified urinary *N*-glycosites were typical sites. Moreover, *N*-glycosites that matched with N-x-T (88% CTRL, 82% ccRCC) occurred more frequently than those that matched with N-x-S (71% CTRL, 69% ccRCC) (Table 2a). The differences between controls and affected subjects emerged by considering the sequence motif around the *N*-glycosites. For the ccRCC samples, we observed a general decrease in the frequency of the N within the consensus regions and a corresponding increase of the frequency within the non-consensus regions (Table 2a).

Subsequently, we investigated the ccRCC samples cohort according to the pT feature, considering the two sub-groups pT1 and pT3, separately. The *N*-glycosites mapping analysis showed a global decrease in the *N*-glycosylation level for the pT1 compared to CTRL group (Table 2b, Figure S1a). In fact, we observed a reduction of 8% when considering the consensus sequence N-x-T, but an increment of the N in the non-consensus sequences (14%) (Table 2b, Figure S1a).

The N as a part of non-consensus motifs was observed to increase even further in the pT3 glycoproteome (29%), suggesting a correlation between an altered glycosylation of non-consensus sequences and ccRCC. We also detected a decrease of glycosylation frequency for the motif N-x-T (-7%) in pT3, despite an overall increment of the total modified asparagine on all the sequences (9%) (Table 2b, Figure S1a).

Considering the distribution of the *N*-glycosylation sites of the peptides present in of the three groups, it is worth noting that the percentages were very similar (Table 2c, Figure S1b); this indicates that the differences observed for all the identified glycosylated peptides are not due to differences in the quantities of the peptides tested. These data support the presence of alterations to the *N*-glycosylation pattern in ccRCC patients compared to healthy subjects.

**Figure 1:**
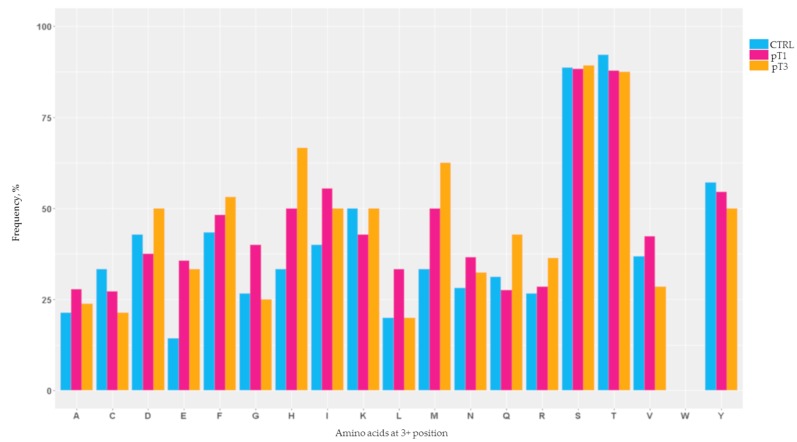
The frequencies of each amino acid in the +3 position in the triplet N-x-x presented in the urinary *N*-glycoproteome calculated for the CTRL, pT1 and pT3 groups. These frequencies were measured considering the identified peptides in all of the three cohorts.

In order to better characterise the *N*-glycosylated peptides, we determined the amino acid composition of the *N*-glycosylation motif. Then, we estimated the frequency of single amino acidic residues present at the +3 position in the modified triplet N-x-x (Figure 1).

Results clearly showed that the consensus sequences N-x-T/S were most frequently modified in both early and advanced cancer conditions (Figure 1).

However, some remarks can also be formulated regarding the frequency of any non-canonical motif. In the pT1 group, for example, a preference for the other amino acids at the +3 position was detectable. Specifically, we recorded an increased frequency of glutamic acid (E), glycine (G), histidine (H), leucine (L), isoleucine (I), methionine (M), asparagine (N) and valine (V). At the same time, we also highlighted a mild inflection for the non-consensus sequence containing cysteine (C), aspartic acid (D), lysine (K) and glutamine (Q) (Figure 1).

In the pT3 group, the non-canonical triplets contained an increased amount of the amino acids D, glutamine (Q) and arginine (R), in addition to those already detected in the pT1 group (Figure 1). Conversely, a consistent decrease in residue V was observed and comparable to both the CTRL and pT1 groups, whilst the amino acids G and L showed a similar level to that of control subjects (Figure 1).

### 2.3. Characterisation of the Urinary N-Glycoproteins: Identification

After characterising the number and distribution of the *N*-glycomodified sites, we evaluated the tryptic peptides identified by nUHPLC-MS/MS in term of proteins identification: 299 different *N*-glycoprotein species were identified with FDR peptide-spectrum matches of 1% and at least one unique peptide (Table S2).

**Figure 2:**
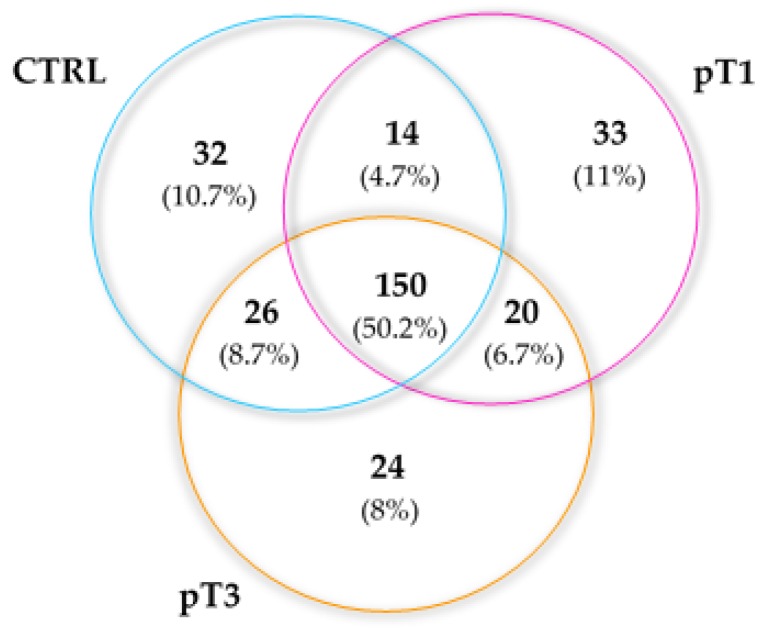
Distribution of the urine *N*-glycoproteins identified by shotgun LC-MS illustrated by a Venn diagram.

Comparing lists of the identified proteins showed that half were shared among the three groups (50.2%). Moreover, we noticed three clusters of proteins that were specific for each group: 11% related to pT1, 8% to pT3 and 10.7% to CTRL (Figure 2).

### 2.4. Characterisation of the Urinary N-Glycoproteins: Functional Analysis

To obtain an overview of the primary subcellular compartments, molecular functions and biological process in which the identified *N*-linked glycoproteins were implicated, we used Cytoscape tool (https://cytoscape.org/last access March 2019) to perform Gene Ontology (GO) function enrichment analysis.

Overall, the most represented subcellular compartments were the extracellular space, lysosomes, membranes, endocytic and extracellular vesicles, which are generally involved in the processes of *N*-glycoprotein synthesis and transport blood components and plasma lipoprotein particles were also significantly enriched (Figure S2a).

Furthermore, many molecular functions that are known to be performed by *N*-glycoproteins were enriched. As shown in Figure S2, the principle clusters affected were binding activity (carbohydrate, chaperone, immune component), extracellular matrix remodelling and receptor activity (Figure S2b).

Finally, the major biological processes that were overrepresented included regulation of vesicle transport, tissue homeostasis, extracellular structure organisation and remodelling, aminoglycan and oligosaccharide metabolisms, endocytosis and blood vessel morphogenesis (Figure S2c).

The principal clusters that emerged for each GO class differed in terms of number of enriched proteins in the controls compared to ccRCC groups. In particular, a substantial increment of those *N*-glycoproteins that compromise a component of immunoglobulin complexes was observed for both early and advanced ccRCC stages whilst an overrepresentation of *N*-glycoproteins related to cellular membrane and immune secretory vesicles could be seen depending upon the pT1 status. For the process of vesiculation, the pT1 stage seemed decrease in terms of endocytic activity and increase in the corresponding secretory activity. This evidence was confirmed either by GO terms, cellular component or biological process (Figure S2a,c).

### 2.5. Characterisation of the Urinary N-Glycoproteins: Quantitative Analysis

A label-free quantitation approach was used to evaluate the relative glycoproteins content present in ccRCC and control urine. Differentially expressed proteins were selected based upon their fold change among the CTRL, pT1 and pT3 groups. We selected those marker-proteins that were detected in two analytical replicates with ≥2 or ≤−2-fold changes (adjusted *p*-value ≤ 0.05).

**Figure 3:**
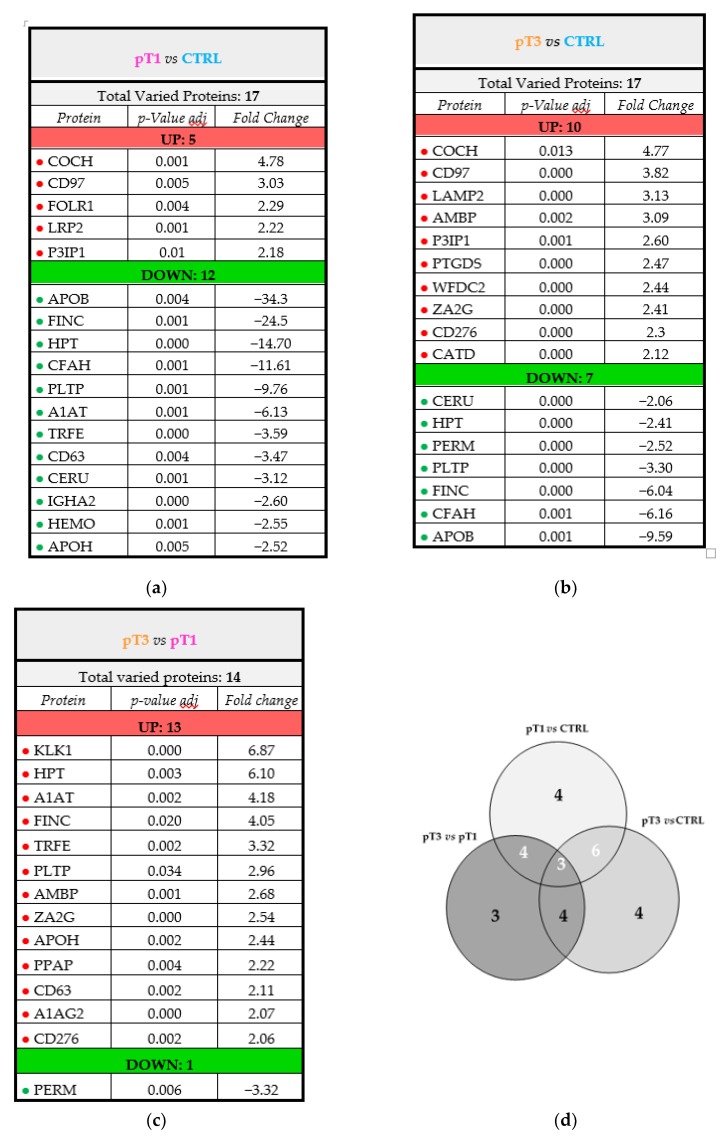
Lists of *N*-glycoproteins significantly varied in the comparisons: pT1 vs. CTRL (**a**), pT3 vs. CTRL (**b**), pT3 vs. Pt1 (**c**). The number of *N*-glycosylated proteins that significantly varied in each group represented by Venn diagram (**d**).

The majority of potential candidate markers were upregulated in the disease *N*-glycoproteome (Figure 3). These included proteins implicated in lipid transport and metabolic processes (e.g., FOLR1, LRP2, PLTP, CATD), immune system processes (e.g., CD97, HPT, A1AT, CD63, PTGDS, CD276) as well as control and maintenance of the cellular shape (e.g., COCH, FINC).

**Figure 4:**
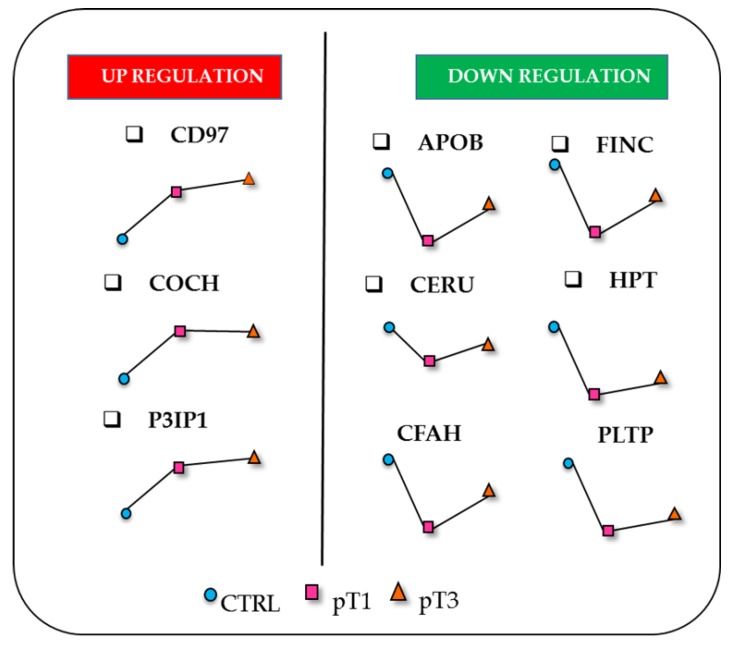
Panel of urinary *N*-glycosylated proteins with the relative abundance trend of each one in the three different sub-cohorts (CTRL, pT1, pT3).

Considering the abundance levels of the significantly dysregulated proteins, we observed a panel of glycoproteins whose specific trend in abundance was related to the stage progression. This panel includes nine proteins of interest, six of which were also identified and quantified in the whole urinary proteome. These nine proteins were differently expressed in the comparisons pT1 vs. CTRL and pT3 vs. CTRL; three of them showed an increasing pattern (CD97, COCH and P3IP1), whilst six presented a decreasing trend (APOB, FINC, CERU, HPT, CFAH and PLTP). It is noteworthy that all the down-regulated glycoproteins showed a considerable decrease in pT1 whilst their levels increased in the pT3 group (Figure 4).

## 3. Discussion

In this study, we detected alterations in the *N*-glycosylation pattern of proteins in ccRCC patient urine relative to healthy subjects. In fact, we observed an increased frequency of glycosylation in non-consensus regions in ccRCC, showing a specific distribution of glycosites related to patients at early (pT1) and advanced (pT3) stages.

Over the years, the *N*-glycan distribution on proteins has been extensively investigated. In fact, many efforts were spent in order to set up the most proficient MS-analytical strategy for mapping the *N*-glycoproteome, enabling the glycoprotein content of different kinds of samples, including cell lines, plants, tissue and bodily fluids to be analysed [25,26,27,28,29,30]. Currently, studies that aim to evaluate the occurrence of *N*-glycosylation within non-consensus sequences in disease conditions are rapidly increasing in number, but the process is still not completely understood. Notwithstanding, acquisition of unconventional *N*-glycosylation sites was described for diverse pathologies, such as follicular and Burkitt’s lymphomas, pancreatic, ovarian, prostatic and gastric cancers [11,31,32,33,34,35].

Some amino acids, such as N, G, C and V were described as part of the *N*-glycosylation motif in several studies [36,37,38] and it was demonstrated that such atypical sites, when glycomodified, play a significant role in biological processes, as well as the canonical counterpart motif T/S [39,40]. As a whole, this suggests that the involvement of glycan macro-heterogeneity may also alter the pathogenesis and progression of ccRCC.

These findings were supported by the isolated *N*-glycoproteins that were identified and quantified in this study. We noted the presence of a group of glycoproteins common to healthy subjects and ccRCC patients at different stages, suggesting the existence of a glycoproteic core of the urinary proteome. In fact, considering the specific role and/or subcellular localisation that the *N*-glycosylation confers to the proteins, it is not surprising that the biological feature of the glycan macro-heterogeneity remains constant, even within a tumour environment [25]. At the same time, each group of samples contained specific proteins and these represented a disease-stage specific signature.

According to their localization, the identified glycoproteins belong to the subcellular compartments, sites of the *N*-glycoprotein formation process. In addition, blood components and plasma lipoprotein particles were significantly enriched since the proteins related to these classes are usually the most abundant ones isolated from the urine [41,42], especially in case of kidney impairment and pathological conditions. Regarding GO classification, as expected, the proteins involved in the immunological pattern were largely enriched, supporting the well-known role of immune response in the onset and progression of ccRCC [43].

In addition, we identified nine *N*-glycoproteins that could be characteristic of the early stage of tumourigenesis, and potentially a glycosignature of the tumour condition. Since ccRCC is a heterogeneous disease, it seems unlikely that only one biomarker will be uniformly elevated. Therefore, a panel of biomarkers may provide more accurate diagnosis than any given single marker.

It is remarkable that PLPT, CD97 and COCH were detected and quantified only after the glycopeptide enrichment, indicating further potential for this approach.

Haptoglobin (HPT), fibronectin (FINC), ceruplasmin (CERU), apolipoprotein-B (APOB), phospholipid transfer protein (PLTP) and complement factor H (CFAH) were down-expressed in urine of RCC patients (Figure 3a,b and Figure 4). Among them, HPT is one of the proteins that is most reported to be affected by oligosaccharide modifications in human malignancies, including ovarian, liver, colon and pancreatic cancers, and its *N*-glycosylation status is different from one type of cancer to another [44,45,46,47,48]. However, all those studies were particularly focused on the *N*-glycan portion of HPT present in serum or plasma samples. To the best of our knowledge, no data that focuses on the *N*-glycosylated isoform of HPT in the ccRCC tumour is currently available. Our result seems to be in contrast with another proteomic study that highlighted an over-secretion of HPT in the urine of ccRCC patients [49]. However, the data are not directly comparable with those of Sadim et al.’s work, where the patients were grouped according with the Fuhrman grade, mixing pT1, 2 and 3, and were of different age compared to our cohort.

Moreover, our data are supported by the findings reported by Bruneel et al. that showed decreased levels of glycosylated HPT in the serum of patients affected by congenital disorders of glycosylation, rare inherited diseases, suggesting that it can be valid as a biomarker with diagnostic relevance for this pathology [50].

A similar consideration can be done for FINC, an extracellular matrix glycoprotein that plays important roles in cellular adhesion and mediation of cell migration and metastasis formation [51,52]. These functions are exploited through *N*-glycosylation modifications, in synergy with integrin-mediated signals [53]. Several studies performed on tissue samples proposed that the overexpression of FINC is an unfavourable prognostic indicator for diverse cancer types, such as breast and pancreatic cancer, nasopharyngeal and neck squamous cell carcinomas [54,55,56]. Regarding RCC, this protein seems to promote cell growth and migration [57]. Moreover, Kondisette et al. investigated the relationship of FINC with the clinical stage of tumour, pinpointed the increase of FINC expression levels in RCC tissue compared to controls, as particularly significant for the early stage condition [58]. In our analysis, we observed a different correlation between the urinary abundance levels of FINC and the cancer stage. In fact, we noted a strong decrease of the *N*-glycosylated FINC levels in the pT1 group (Figure 3a and Figure 4), both for the total protein and the glycosylated form. In light of this, it is arguable that FINC could be retained by cancer cells, and not secreted in urine. On the other hand, a sort of balance between the *N*-glycosylated and the non-glycosylated isoform of FINC could exist and may occur in order to decrease the expression of *N*-glycosylated FINC, in favour of the non-glycosylated form, and may represent a fundamental equilibrium in the early process of ccRCC tumorigenesis [59].

The glycoprotein CERU is normally synthesised in the liver and is involved in different cellular processes [60]. Several studies have demonstrated that CERU expression is required for the growth and survival of tumours. In fact, CERU protein levels were increased in various forms of human tumours, including breast (saliva and plasma), bile duct (tissue microarray), oral and gastrointestinal tract cancers (blood) [61,62,63,64]. On the contrary, CERU abundance was decreased in urine of the HER2-enriched subtype of breast cancer with respect to healthy controls [65]. Concerning ccRCC, it is reported that the expression of the *CERU* gene can distinguish a subset of RCC patients with poor outcome, independently from the known genetic aberrations [66]. In addition, a quantitative proteomic study associated the high serum levels of CERU with the histopathological classification of stage and/or grade of RCC [67]. Moreover, high levels of CERU were found in ccRCC patients at the level of urinary exosomes, a specific compartment of urine [68]. However, no previous studies that focused specifically on the role of the *N*-glycosylation of this protein in ccRCC are present in the literature.

Known as the primary apolipoprotein, APOB plays a key role in lipid transport, lipoprotein metabolism and the recognition of lipoprotein receptors. It is the major constituent of the atherogenic particles and its plasma concentration represents a useful marker for cardiovascular risk. Moreover, increasing evidence shows that high concentrations of plasma APOB are also associated with poor prognosis in patients with hepatocellular carcinoma [69] and breast cancer [70]. High levels of APOB in urine were proposed as a potential marker and a prognostic factor for bladder cancer [71]. The analysis of tissue microarrays highlighted the cytoplasmic expression of APOB in ccRCC, indicating the storage of lipoproteins rather than lipids [72]. Very recently, the APOB/A1 ratio in preoperative blood sample was proposed as an independent prognostic factor in metastatic renal cell carcinoma [73]. Here, we detected APOB in urine, focusing on its glycosylated isoform (Figure 3a,b and Figure 4). It is of particular relevance considering that there is a specific influence of *N*-linked glycosylation on LDL pathways and lipid metabolism [74].

Interestingly, among the candidate glycomarkers, there is another protein involved in lipoprotein metabolism. PLTP is a non-specific lipid transfer protein [75]. Remarkably, this is the first report of PLTP being present in urine and of its association with RCC. It seems to play a role in lipoprotein assembly, which also involves APOB, although there is no evidence of any direct interaction [76]. In this regard, since that both the proteins are part of the lipoprotein metabolism, the simultaneous reduction of PLTP and APOB in the urine of ccRCC patients could make sense (Figure 3a,b and Figure 4). Moreover, the differential expression of PLTP is documented in multiple types of tumours, including prostate, ovarian, breast, lung carcinoma and glioma. In particular, the study performed on brain tissue of glioma patients showed a correlation between PLTP expression and the tumour grade [77].

The plasma glycoprotein CFAH is the main regulator of the alternative complement pathway, inhibiting the activation of the complement system and thus protecting host cells [78]. Given its role, CFAH alterations can have serious implications and cause various diseases, including several cancers, such as non-small cell lung cancer, ovarian cancer, and colon cancer [79,80,81]. In particular, in human lung cancer tissues, CFAH over-expression was associated with a shorter survival time [82]. There are no relevant data regarding the expression of CFAH in relation with RCC, nor in tissue, nor in biological fluid studies. As hypothesised for FINC, cancer cells could modulate the secretion of CFAH in order to maintain high levels in the tumour microenvironment as a mechanism of protection.

Among the over-expressed proteins, PIK3IP1 is a cell-surface glycoprotein reported as a negative regulator of the PI3K pathway [83]. The transcriptional regulation and expression of PIK3IP1 directly affects the PI3K signalling in tumorigenesis. In fact, the downregulation of this inhibitor contributes to increased tumour growth [84], whilst its over-expression in mouse hepatocytes leads to the suppression of hepatocyte carcinoma development [85]. In this context, it is noteworthy that this protein was over-represented in the urine of ccRCC patients (Figure 3a,b and Figure 4). In this case, the assumption made for FINC, APOB and PLTP would be the opposite: cancer cells eliminate more PIK3IP1 protein in the urine, as a mechanism to down-regulate its expression, in order to survive.

Finally, we found CD97 and COCH over-expression in RCC patient urine (Figure 3a,b and Figure 4). There is currently no evidence of an association with RCC. CD97 is an adhesion G protein-coupled receptor (GPCR) that is expressed on multiple hematopoietic cell types. Favouring the migration and invasive properties of cancer cells, it is involved in various non-hematopoietic malignancies including breast, thyroid, gastric, and prostate carcinoma, as well as glioblastoma. Furthermore, the role of CD97 was explored in acute myeloid leukaemia and was also related to the PI3K/Akt pathway [86]. COCH is a protein of the extracellular matrix (ECM) that is expressed prevalently at the cochlea level and is associated mainly with hearing defects [87]. Its function is not completely understood, and it is presented in numerous heterogeneous isoforms because of *N*-glycosylation modification and proteolytic cleavage.

To conclude, we also investigated the gene expression of our putative glycomarkers in comparison with the Human Protein Atlas database (www.proteinatlas.org). We considered the mRNA expression in the cell-enriched group in order to verify the renal tissue specificity of the urinary dysregulated proteins identified in our study. Different mRNA expression levels of CD97, CERU, CFAH, COCH, FINC, HPT, PLTP and P3 IP1 were detected and confirmed in diverse cell lines related to the urinary tract, including normal human embryonic renal cell line (HEK 293), human embryonal carcinoma cell line (NTERA-2), normal human renal proximal tubule epithelial cell line (RPTEC/TERT1), human hypertriploid renal carcinoma cells (786-O cells), human renal cell line (Caki-1), human bladder cancer cell line (RT4) and human prostate cancer cell line (PC-3). To our knowledge, no data concerning the gene expression of APOB in other cell line models suitable for the in vitro study of ccRCC are available.

## 4. Materials and Methods

### 4.1. Urine Collection and Processing

Forty-five patients, 30 ccRCC and 15 non-RCC, were enrolled between 2011 and 2016 at San Gerardo hospital (Monza, Italy). All participants gave their informed consent prior to sample collection. Study protocols and procedures were approved by the local ethic committee (Comitato Etico Azienda Ospedaliera San Gerardo, Monza, Italy, BPCR 24-02-2011) and analysis were carried out in agreement with the Declaration of Helsinki. Second morning midstream urine before total or partial nephrectomy (for ccRCC patients) was collected in sterile urine tubes. The collected samples were cleared by centrifuging at 4 °C at 1000× *g* for 10 min to remove cells and debris and stored at −80 °C until the day of the analysis.

### 4.2. Sample Population

Three urine pools were prepared (15 subjects each), one for the CTRL, one for ccRCC at pT1 and one for ccRCC at pT3. The stage stratification was confirmed by radiological evaluation and pathological assessment of the surgical specimens. Each patient contributed to the pool sample with an equal amount of proteins (70 µg).

### 4.3. Urinary Proteins Digestion

Two milliliter of each of the urine samples was defrosted and urinary proteins were precipitated by nine volumes of cold 90% ethanol and pelleted at 3500× *g* for 30 min [88]. After drying, proteins were dissolved in bidistilled water, and protein concentration was assessed by BCA assay (Microplate BCA™ protein Assay Kit, Thermo Scientific, Waltham, MA, USA), using BSA as standard.

Approximately 400 μg of pooled proteins were digested following the FASP protocol, as already described [89]. Briefly, proteins were first reduced by incubation with 50 mM DL-dithiothreitol (Sigma Aldrich, Switzerland) and alkylated for 30 min with iodoacetamide 100 mM (Sigma Aldrich, Switzerland). Then, they were digested overnight on 30 kDa filters (Amicon Ultra-500 30 kDa, Millipore, New York, NY, USA) adding trypsin from porcine pancreas (Proteomics Grade, BioReagent, Dimethylated) in a ratio 1:100 to the initial protein concentration. After repeated washing of the filter, the eluted peptides were collected and lyophilised. The resulting peptides were resuspended in steril-filtered water (Sigma Aldrich, Buchs, Switzerland) and their concentration was determined by nanodrop spectrophotometer (Thermo Scientific^TM^).

### 4.4. Urinary N-Glycopeptides Enrichment and Deglycosylation

After the whole urinary proteome digestion, the *N*-glycopeptides were enriched by the lectins-capture strategy, using a FASP-based method (so-called *N*-Glyco-FASP) that was first described by Zielinska et al. [25]. Briefly, a solution containing a pool of three different types of lectins, concavalin A (ConcA), wheat germ agglutinin (WGA) and agglutinin RCA_120_ (Sigma Aldrich) was mixed with approximately 100 µg of digested peptides with a mass proportion of 1:3. The mixture was loaded on a new 30 kDa filter unit (Amicon Ultra-500, 30 kDa, Millipore) and the lectins-glycopeptides incubation was performed at 4 °C for 2 h. Then, the unbound peptides were eluted by four steps of centrifugations at 14,000× *g*, each one for 10 min. The captured peptides underwent overnight deglycosylation with PNGase F (Roche, New York, NY, USA) at 37 °C. The deglycosylated peptides were eluted with another centrifugation series (14,000× *g*, 10 min), lyophilised and stored at -20 °C, until the following MS analysis.

### 4.5. Mass Spectrometric Analysis

Two microgram of *N*-glycopeptides was injected into nanoHPLC and separation was performed using 50 cm nanocolumn (Dionex, ID 75 μm, Acclaim PepMap100, C18, 2 μm, Sunnyvale, CA, USA). The separation was performed at 40 °C and at a flow rate of 300 nL/min, using a multistep 4-h gradients that increased the percentage of B from 4 to 66% in 204 min (mobile phase A, used for nano-pump H_2_O w/0,1% formic acid and whilst mobile phase B was composed of 80:20 ACN: H_2_O w/0.08% FA). An Impact HD™ Ultra High Resolution-QqTOF (Bruker Daltonics, Bremen, Germany), equipped with a nanoBoosterCaptiveSpray™ ESI source (Bruker Daltonics) was used in the Data-Dependent-Acquisition mode. The MS/MS data were acquired by targeting precursor ions (*m/z* 300–2000 range) with a charge state between +2 and +5. The fragmentation was performed by collision-induced dissociation (CID). Both the MS scans and MS/MS data were recorded as line spectra based on centroided data.

Internal calibration, using a lock mass of *m/z* 1221.9906, and a calibration segment based on a 10 mM sodium formiate cluster solution (15 min before each run) were used to correct the raw MS and MS/MS data Compass DataAnalysis v4.1 software (Bruker Daltonics) was used to calibrate, deconvolute and convert the acquired raw data prior to protein identification and quantification.

### 4.6. Data Processing

#### 4.6.1. Protein Identification

Mascot (v 2.4.1, Matrix Science, London, UK) was used for protein identification. Trypsin was chosen as the enzyme and the number of missed cleavages was set to 1. The peptide charge was set to 2+ and 3+, and the peptide tolerance and MS/MS tolerance were 20 ppm and 0.05 Da, respectively. Cysteine carbamidomethylation was set as fixed modification, whilst methionine oxidation and asparagine deamidation were used as variable modifications. Swiss-prot was used as database (accessed May 2017, 555.594 total entries). The maximum false discovery rate (FDR) for peptide spectral match was set to 1%, using percolator algorithm and a minimum of one sequence-unique peptides was required for identification. Proteins of interest were analysed for cellular component, molecular functions and biological processes with ClueGO v2.5, Clupedia v1.5 and the Cytoscape tools [90].

#### 4.6.2. Bioinformatics and Statistical Analysis

Progenesis QI for proteomics v.2.0.5387.52102 (Nonlinear Dynamics, Newcastle, UK) was used for the label-free protein quantification [91]. Data were imported as centroided data and automatic alignment with additional manual adjustment were performed to maximise the overlay between runs. Peak picking was achieved with a default sensitivity, a minimum peak width of 0.2 min and maximum charge of 8. Normalisation was applied using Progenesis software and calculated over all peptide ions by a global scaling factor between the samples based on selected reference.

Peptides were identified using an in-house Mascot search engine as described previously. Only non-conflicting peptides were used for the relative quantification. Protein abundance was calculated using the sum of all unique normalised peptide ion abundances for each specific protein in each single analysis. Statistical analyses for quantitative evaluation were performed using the open-source R software v.3.5.0. For the comparison between the different sample cohorts in terms of *N*-glycoprotein abundance the Anova test was used and a post-hoc Tukey test, with Benjamini and Hochberg adjustment, was applied for pairwise comparisons. For each test, the level of significance was set equal to 0.05. Proteins with at least two-fold changes were considered differentially regulated. For *N*-glycomapping characterisation of the glycopeptides, all the potential *N*-glycosylation sites were investigated in order to recognise and count all the deamidated asparagine present in sequences obtained from Uniprot database (http://www.uniprot.org.), using R language. Peptides that were found to be already deamidated in the whole proteome were excluded from the further analysis.

## 5. Conclusions

Despite recent developments in our understanding of glycosylation processes, an in-depth comprehension of the *N*-glycoproteome in ccRCC is still lacking. *N*-glycoproteins are involved in numerous fundamental processes that occur during cancer, such as tumour cell invasion, cell-matrix interactions, tumour angiogenesis, immunomodulation and metastasis genesis and propagation. It is well-known that unusual glycosylation of the protein causes severe defects in protein localisation, trafficking, cellular adhesion and transduction pathways in disease conditions, which may lead to valuable diagnostic markers.

To the best of our knowledge, our study is the first that thoroughly investigates the urinary *N*-glycoproteome in patients with early and advanced ccRCC conditions and compares this with non-ccRCC subjects. We evaluated the urinary glycoprotein content both qualitatively (*N*-glycosites distribution) and quantitatively (*N*-glycoprotein expression) and highlight a specific distribution of glycosites that is related to patients at early (pT1) and advanced (pT3) stages. These alterations suggest that the *N*-glycosylation of motifs different from the most relevant N-x-T/S may have role in cancer status, as already shown in other studies. In fact, it is well known that some of the unconventional glycosylations are carried out by unique classes of glycosyltransferase enzymes. Therefore, it could be highly interesting to verify the existence of these *special enzymes* in a future study and explore the mechanisms that they adopt to recognise both consensus and non-consensus motifs, performing a targeted study in ccRCC cells lines or tissue biopsies.

Additionally, our investigation highlights the presence of a group of nine urinary *N*-glycoproteins with a specific abundance trend that could constitute a specific disease-related glycosignature and may be able to underlined the transition from early to the advanced stage. These dysregulated proteins may have important implications in the pathogenesis of this renal malignancy, potentially playing a role that is different from their corresponding non-glycosylated isoform in the process of tumourigenesis and may be useful for future diagnostic applications. Finally, our data encourage further investigations that target the in-depth characterisation of the *N*-glycans attached to the deregulated proteins identified in order to determine the precise role of this important PTM in the onset and development of ccRCC.

## Figures and Tables

**Table 1:** Clinicopathological data regarding the patients enrolled in the study.

Group	# of Patients	Gender (Male-Female)	Age Mean (Range)	Tumour Dimension (cm)
CTRL	15	10–5	57.9 (39–77)	/
pT1	15	8–7	67.8 (42–82)	4
pT3	15	12–3	68.9 (45–81)	12

**Table:** (**a**) Comparison of the *N*-glycosylation distribution (%) between the CTRL and ccRCC patients cohorts.

*N*-glycosylated Asparagine Distribution	%	
	CTRL	ccRCC
N/N tot	21	21
N in ncSeq/N ncSeq tot	5.6	6.8
N in cSeq/N cSeq tot	81	77
N-x-T/N-x-T tot	88	82
N-x-S/N-x-S tot	71	69

**Table:** (**b**) Comparison of the *N*-glycosylation distribution (%) between CTRL and pT1 and CTRL and pT3 patients cohorts, measured by considering all the identified peptides, shared and not shared in the three groups.

%				%		
CTRL	pT1	Variation		CTRL	pT3	Variation
21	19	−10	N/N tot	21	23	9
5.6	6.4	14	N in ncSeq/N ncSeq tot	5.6	7.2	29
81	75	−7	N in cSeq/N cSeq tot	81	79	−2
88	81	−8	N-x-T/N-x-T tot	88	82	−7
71	66	7	N-x-S/N-x-S tot	71	73	3

**Table:** (**c**) Comparison of the *N*-glycosylation distribution (%) between CTRL and pT1 and CTRL and pT3 patients cohorts, determined by taking into account the identified peptides common to all three groups.

%				%		
CTRL	pT1	Variation		CTRL	pT3	Variation
66	66	0	N/N tot	66	66	0
31	37	19	N in ncSeq/N ncSeq tot	31	37	19
91	88	−3	N in cSeq/N cSeq tot	91	88	−3
92	88	−4	N-x-T/N-x-T tot	92	88	−4
89	88	−1	N-x-S/N-x-S tot	89	89	0

ncSeq: non-consensus sequence; cSeq: consensus sequence; N: asparagine glycomodified; N: asparagine glycomodified and non glycomodified.

## Supplementary Materials

The following are available online at https://www.mdpi.com/2072-6694/12/1/239/s1, Figure S1: Trend distribution of the *N*-glycosylation sites of the urinary glycoproteins, Figure S2: Gene ontology analysis of the *N*-glycoproteins identified from all sample groups by LC-MS , Table S1: List of *N*-glycoproteins identified in the CTRL, pT1, pT3 groups, Table S2: List of *N*-glycomodified peptides in the CTRL, pT1, pT3 groups.
