# Supplementary material for: In-Depth Mapping of the Urinary N-Glycoproteome: Distinct Signatures of ccRCC-related Progression
Source: Cancers (Basel). 2020 Jan 18;12(1):239. doi: 10.3390/cancers12010239 (PMC7016614; doi:10.3390/cancers12010239)
Supplement: Supplementary file 1 [file cancers-12-00239-s001.zip › Figure S1.pptx]

## Slide 1
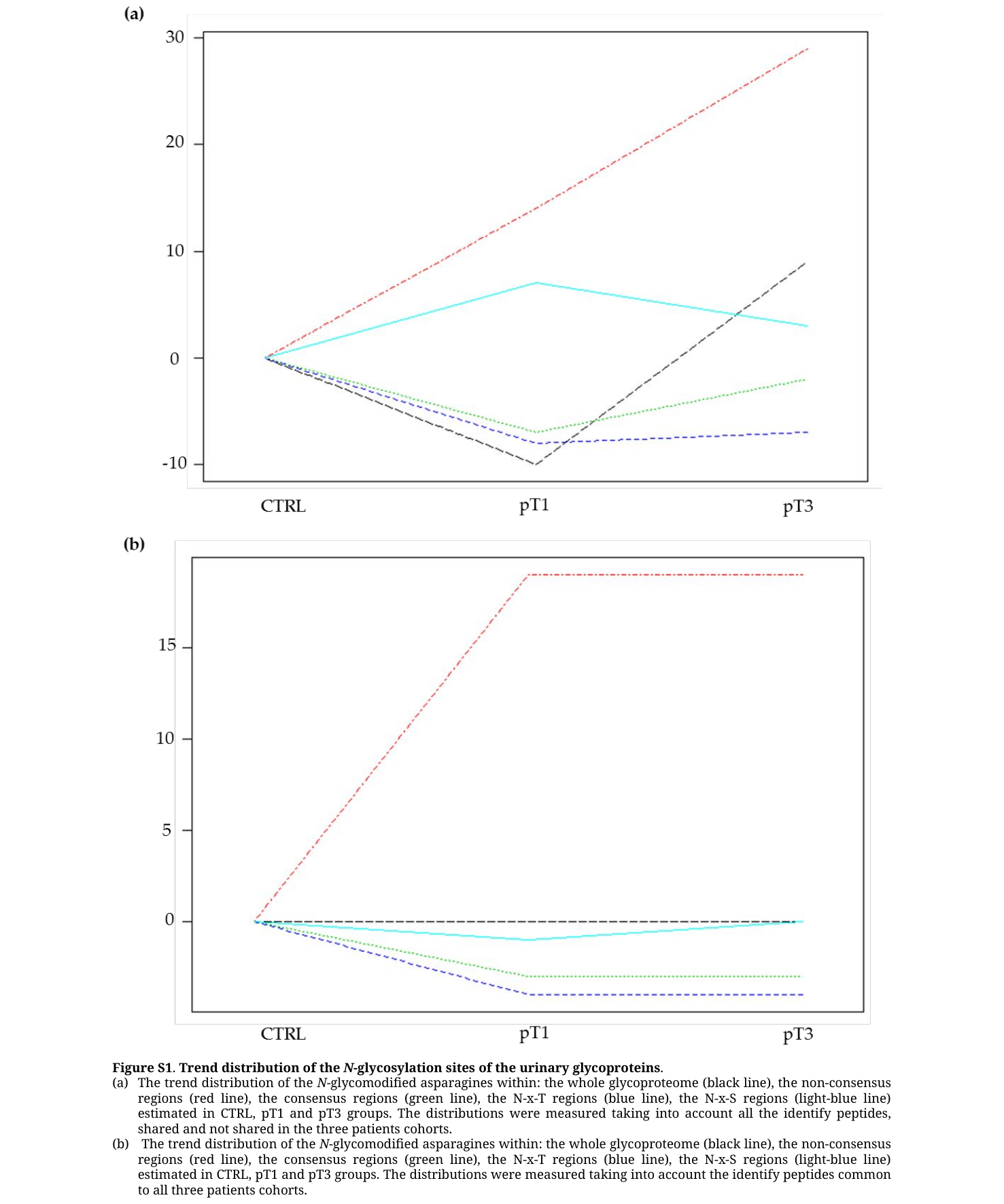

Figure S1. Trend distribution of the N-glycosylation sites of the urinary glycoproteins.
The trend distribution of the N-glycomodified asparagines within: the whole glycoproteome (black line), the non-consensus regions (red line), the consensus regions (green line), the N-x-T regions (blue line), the N-x-S regions (light-blue line) estimated in CTRL, pT1 and pT3 groups. The distributions were measured taking into account all the identify peptides, shared and not shared in the three patients cohorts.
 The trend distribution of the N-glycomodified asparagines within: the whole glycoproteome (black line), the non-consensus regions (red line), the consensus regions (green line), the N-x-T regions (blue line), the N-x-S regions (light-blue line) estimated in CTRL, pT1 and pT3 groups. The distributions were measured taking into account the identify peptides common to all three patients cohorts.
