# Supplementary material for: In-Depth Mapping of the Urinary N-Glycoproteome: Distinct Signatures of ccRCC-related Progression
Source: Cancers (Basel). 2020 Jan 18;12(1):239. doi: 10.3390/cancers12010239 (PMC7016614; doi:10.3390/cancers12010239)
Supplement: Supplementary file 1 [file cancers-12-00239-s001.zip › Figure S2.pptx]

## Slide 1
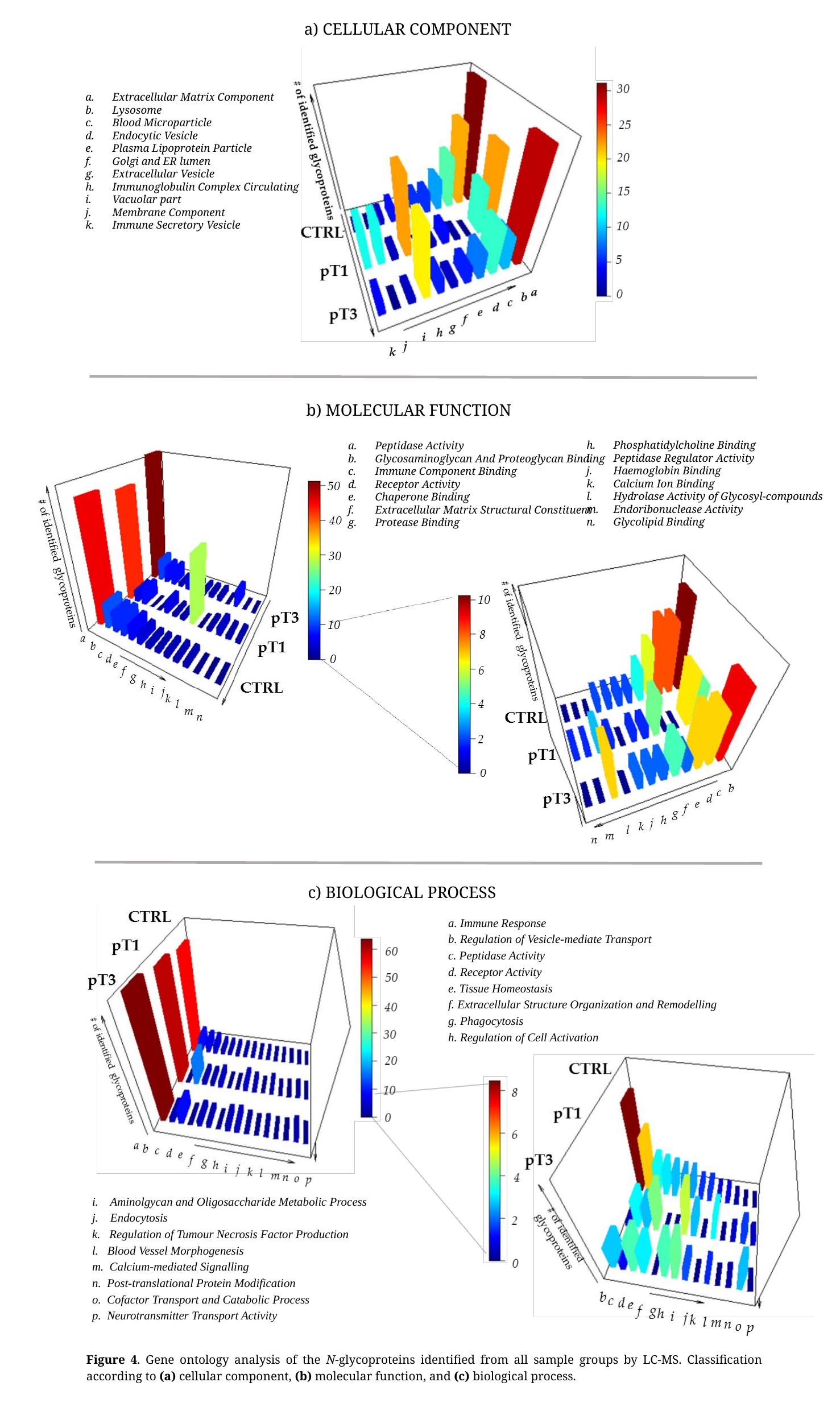

a) CELLULAR COMPONENT
Extracellular Matrix Component
Lysosome
Blood Microparticle
Endocytic Vesicle
Plasma Lipoprotein Particle
Golgi and ER lumen
Extracellular Vesicle
Immunoglobulin Complex Circulating
Vacuolar part
Membrane Component
Immune Secretory Vesicle
b) MOLECULAR FUNCTION
Phosphatidylcholine Binding
Peptidase Regulator Activity
Haemoglobin Binding
Calcium Ion Binding
Hydrolase Activity of Glycosyl-compounds
Endoribonuclease Activity
Glycolipid Binding
Peptidase Activity
Glycosaminoglycan And Proteoglycan Binding
Immune Component Binding
Receptor Activity
Chaperone Binding
Extracellular Matrix Structural Constituent
Protease Binding
c) BIOLOGICAL PROCESS
a. Immune Response
b. Regulation of Vesicle-mediate Transport
c. Peptidase Activity
d. Receptor Activity
e. Tissue Homeostasis
f. Extracellular Structure Organization and Remodelling
g. Phagocytosis
h. Regulation of Cell Activation
i. Aminolgycan and Oligosaccharide Metabolic Process
j. Endocytosis
k. Regulation of Tumour Necrosis Factor Production
l. Blood Vessel Morphogenesis
m. Calcium-mediated Signalling
n. Post-translational Protein Modification
o. Cofactor Transport and Catabolic Process
p. Neurotransmitter Transport Activity
Figure 4. Gene ontology analysis of the N-glycoproteins identified from all sample groups by LC-MS. Classification according to (a) cellular component, (b) molecular function, and (c) biological process.
